# Process and Implementation Elements of Measurement Feedback Systems: A Systematic Review

**DOI:** 10.1007/s10488-023-01325-3

**Published:** 2023-12-28

**Authors:** Kristian Rognstad, Thomas Engell, Krister Fjermestad, Tore Wentzel-Larsen, John Kjøbli

**Affiliations:** 1 Regional Center for Child and Adolescent Mental Health, Eastern and Southern Norway, Oslo, Norway; 2 Department of Psychology, University of Oslo, Oslo, Norway; 3 Norwegian Centre for Violence and Traumatic Stress Studies, Oslo, Norway; 4 Department of Education, University of Oslo, Oslo, Norway

**Keywords:** Measurement feedback systems, Patient reported outcome measures, Feedback informed treatment, Implementation, Adherence, Fidelity

## Abstract

Measurement feedback systems (MFS) can help guide treatment and improve clinical outcomes. Studies of MFS are heterogeneous both in execution and results, and the effects of MFS seem restricted by limited attention to process and implementation elements and by limited adoption by health professionals. The current systematic review mapped the use of process and implementation elements in MFS studies. An overview of therapists’ use of and attitudes toward MFS is provided. Three-level meta-analyses were used to test theoretically informed process and implementation elements as moderators of the effects of MFS. Hypotheses and general propositions from Clinical Performance Feedback Intervention Theory (CP-FIT) were used to organize the elements of the studies and were used as moderator variables. Previous studies on MFS interventions have had a limited focus on implementation efforts and process elements that may increase the effects of MFS and their use among therapists. Efforts have sparsely been made to reduce barriers to MFS use, and several studies have reported limited engagement with MFS among therapists. Therapists’ attitudes toward MFS, feedback, or standardized measures were heterogeneously reported, making data synthesis challenging. Identified process and implementation elements were not significantly associated with effect sizes in the studies and the results did not support the propositions of CP-FIT. The lack of statistically significant associations may be due to limited reporting of details about process and implementation aspects. More research designed to test hypotheses regarding process and implementation elements is needed to improve the use and effects of MFS. Future studies should aspire to report findings in a manner that allows for an understanding of the implementation process and therapists’ adoption of these systems.

**Supplementary Information:**

The online version contains supplementary material available at 10.1007/s10488-023-01325-3.

## Introduction

Patients often leave therapy for mental health disorders without experiencing symptom relief or better functioning (Warren et al., 2010; Wolpert, 2016). Concomitantly, the magnitude of the effect of therapy has not increased in clinical trials over the last decades (Weisz et al., 2017), illustrating the need for new approaches to enhance treatment effects. One such approach is the use of measurement feedback systems (MFS) to guide therapy by informing therapists and/or others involved about changes for patients. In several studies, MFS have had positive effects on clinical outcomes (e.g., Amble et al., 2014; Brattland et al., 2018), and recent meta-analyses have presented small positive overall effect estimates (de Jong et al., 2021; Rognstad et al., 2022). Still, the effect of MFS seems hampered by a lack of attention to the process and implementation elements necessary for its success (Bickman et al., 2016; Kendrick et al., 2016).

Feedback-informed therapy involves the continuous and systematic collection of information about the patient. The use of standardized measures throughout the course of treatment is meant to improve care by ensuring better informed patients, health care providers, and/or other stakeholders. Progress, or lack thereof, can be tracked throughout treatment, which helps clinicians evaluate, adjust, and tailor the treatment course to the individual patient. Still, the implementation of MFS can be complicated.

Several systematic reviews point out that there is substantial heterogeneity in the results from MFS studies (Gondek et al., 2016; Ishaque et al., 2019; Rognstad et al., 2022). This is likely due in part to implementation issues and therapists’ reception of MFS interventions (Kendrick et al., 2016). In a narrative review, Lewis et al. (2019) present a 10-point research agenda for measurement-based care (MBC), which is a form of care that relies on MFS to inform decision making. They point to the need for the development of a criterion standard method for monitoring fidelity to MFS and understanding elements that facilitate fidelity. Identifying important process and implementation elements that facilitate fidelity to MFS can inform the development of fidelity standards and assessments. Degree of implementation has been shown to moderate the effectiveness of MFS, and a dose–response effect has been found showing a larger treatment effect in individual cases where the MFS was more frequently used (Bickman et al., 2016). Brattland et al. (2018) reported increased effects of MFS over time and proposed that this might be due to continued implementation efforts such as regular MFS training and supervision throughout the trial period of about 4 years. Also, based on a systematic review of qualitative studies, Brown et al. (2019) developed a comprehensive theoretical framework to describe how clinical performance feedback systems such as MFS work as interventions. This Clinical Performance Feedback Intervention Theory (CP-FIT) encompasses processes and mechanisms related to implementing and using MFS theorized as determinants of success. CP-FIT provides a framework for a theory-informed review of elements that are important for the delivery and implementation of MFS.

### Clinical Performance Feedback Intervention Theory

CP-FIT was developed to describe causal pathways in audit and feedback processes. It proposes that feedback is a cyclical process comprising goal setting; data collection and analysis; feedback; recipients’ interaction with feedback; and perception and acceptance of the feedback resulting in intention, behavior, and clinical performance improvement (Brown et al., 2019). CP-FIT further states that feedback will be less effective if any of these individual processes fail.

CP-FIT identifies variables related to the feedback, recipients, and context that influence how successful feedback interventions are. The theory suggests that feedback needs to be clinically meaningful to the recipients, automatically collected and analyzed, actively delivered, easily comprehended, and target goals within the recipient’s control. Also, feedback recipients should feel ownership and control over the interventions, as well as have a positive view of feedback. Further, CP-FIT suggests that feedback interventions should be implemented with leadership and opportunities to discuss feedback with peers and that they should not be time and energy-consuming.

Three general propositions are put forth by CP-FIT to summarize 42 more specific hypotheses. The three general propositions are: (1) Capacity limitations—less taxing feedback interventions will work better due to therapists’ and organizations’ finite capacity; (2) Identity and culture—feedback interventions better aligned with therapists’ and organizations’ beliefs about patient care will be more successful; and (3) Behavioral induction—feedback interventions that directly support clinical behavior will be more effective.

### Practice, Process, and Implementation Elements

Different approaches to identifying common practice elements have been adopted to identify potentially active ingredients and mechanisms of change in treatment programs (Chorpita et al., 2005; Helland et al., 2022; Kjøbli et al., 2023; Kvamme et al., 2022; Leijten et al., 2019). A “practice element” is any “discrete clinical technique or strategy used as part of a larger intervention plan” (Chorpita et al., 2005). Identifying practice elements can help with “unboxing” interventions and implementations and, through different methods, with finding the elements that cause change or are highly influential (Engell et al., 2023).

In addition to identifying effective practice elements in MFS, the circumstances needed for MFS interventions to be effectively used should be mapped. Whereas a practice element is what people do, a process element is how people do things or how things unfold or emerge (Engell et al., 2023). Implementation elements, however, are what makes people do what things, and implementation elements can include discrete implementation strategies (Powell et al., 2015), implementation determinants (Nilsen & Bernhardson, 2019), implementation competencies (Metz et al., 2021), or any other relevant part of the implementation processes (Engell et al., 2023). While process elements tend to describe delivery forms and contexts, implementation elements tend to describe facilitating actions such as ongoing training or technical support, and motivations and states such as attitudes towards the intervention or readiness for implementation. Implementation elements can also be the implementation competencies such as abilities to interact with service providers, communicate research findings, improve research-practice partnerships (Metz et al., 2021) or competence in implementation facilitation, context assessment or knowledge of implementation theories, models, and frameworks (Bührmann et al., 2022). Such aspects of interventions and implementations may be necessary or catalyzing conditions for effects to occur or emerge (Engell et al., 2023). This study will focus on process and implementation elements.

### Process and Implementation of MFS

Therapists are often reluctant to engage with feedback data due to both philosophical reasons (e.g., belief that assessment disrupts the flow of treatment or that the measures do not address relevant constructs) and practical implementation barriers (Cooper et al., 2021). Receiving negative feedback can be intimidating and may cause therapists to lose optimism (Lambert et al., 2019). In a systematic review, the most common barriers to therapists’ use of feedback were the perceptions that the relevance of feedback is limited, data collection and synthesis are time-consuming, and MFS is intrusive in the context of clinical practice (Gelkopf et al., 2022).

MFS have been implemented in a variety of contexts and frameworks. The systems themselves are also heterogeneous. Each system comprises various elements, with, for instance, different surveys for patients and differences in the presentation of the collected information to the therapists. Lyon et al. (2016) describe several capabilities and characteristics of existing MFSs, thus providing a useful mapping of commonalities and differences in existing systems. However, there is limited research on how specific elements/components are associated with effective implementation and use of MFS. A system’s capabilities are no guarantee that it will be used—merely granting access does not mean the feedback is read. Further, reading the feedback does not guarantee understanding or clinical use of the information. Identifying the elements that can reduce barriers and increase use is important. Some aspects in this regard will be found outside of the system per se, e.g., in support systems for understanding the data, for collection of the data, ease of use, and efforts to build a culture for feedback use.

#### Process Elements of MFS

Computer-based systems for data collection and analysis could ease the burden on service providers. Systems that gather data outside of therapy sessions may be less disruptive to therapy, and the brevity of the measures may reduce stress for respondents. Automatic data synthesis, including scoring systems and individual graphs, can facilitate interpretation and use (Gelkopf et al., 2022). Easy access to data presented in an understandable fashion should increase use and effects; elaborate logins and unexplained raw data may produce difficulties in access and reduce intentions to use the data. MFS can provide therapists with scores on the scales used, graphs illustrating change over time, typologies of patients, and warning signals for not-on-track patients. The accuracy of predictions will increase if they are based on a comparison with normative patient progress data.

#### Implementation Elements of MFS

Implementers prioritize training, guidance, standby support, and manuals for the use and interpretation of the feedback data to different degrees. Such resources may help therapists understand how to use the data and its benefits. Well-informed therapists are also more likely to explain what MFS do and the reasons for using them to patients, who report uncertainty about their purpose and usage when encountering MFS (Börjesson & Boström, 2020). Studies differ in terms of whether the therapists participated voluntarily or whether the MFS was mandatory for the entire institution, both of which are likely to influence “ownership” and adherence. Feedback could be accompanied by discussion groups or supervision to help therapists understand data and revise treatment plans. Creating a “culture of feedback,” where opinions about the process and the outcome of services are welcomed and likely to have an impact on the nature and quality of services, may increase the use of feedback (Miller et al., 2016). MFS range from those that provide therapists with feedback data to those that provide suggestions for resolving identified problems. One meta-analysis indicated an additional effect in a subgroup of not-on-track patients from adding therapy suggestions through Clinical Support Tools (CST) to the MFS (Lambert et al., 2018).

In summary, a wealth of different strategies may be applied to improve the adoption and effects of MFS. More efficient systems may be created by identifying the elements that contribute, and those which do not contribute, to desired therapy outcomes. A systematic review and meta-analyses of theoretically informed process and implementation elements in MFS are thus needed to explore explanations for the heterogeneity in effects and large variations in feedback use and identify elements likely to improve the implementation and effectiveness of MFS.

### The Current Review

The current review aimed to identify process and implementation elements in the MFS literature to provide an overview of the state of the field. Furthermore, we tested the impact of these elements on the effect of MFS by applying them as moderators in a meta-analysis. We also intended to map therapist use of feedback and therapist attitudes toward feedback use and apply them as moderators for the effect of the MFS.

Three central propositions of the CP-FIT informed our operationalization of process and implementation elements in MFS, i.e., capacity limitation, identity and culture, and behavioral induction. We hypothesized that measures taken to limit how taxing the systems are on clinicians (capacity limitation), ensure positive attitudes and a sense of ownership of the feedback (identity and culture), and support clinicians in comprehension and initiation of alternative treatment plans in response to feedback (behavioral induction) would increase use and effects of MFS. Identified elements were used to consider whether the prerequisites for well-functioning feedback systems according to each of CP-FIT’s 42 specific hypotheses were fulfilled. The scores on each hypothesis were then summarized in the three central propositions of the model.

## Method

The current article is based on an update and secondary analyses of a review preregistered in PROSPERO (Code CRD42021240379) and follows the Preferred Reporting Items for Systematic Reviews and Meta-analyses (PRISMA) guidelines (Page et al., 2021). The review included both cluster-randomized and individually randomized studies with control conditions that were similar to the active condition except for the MFS tested. Participants were in treatment for common mental health problems (depression; mixed anxiety and depression; and specific anxiety disorders, such as generalized anxiety disorder (GAD), phobias, obsessive–compulsive disorder (OCD), panic disorder, and post-traumatic stress disorder (PTSD)) in any primary care, out- or inpatient therapy, or multidisciplinary mental health care setting.

### Inclusion Criteria

- Randomized controlled trials, both cluster-randomized and randomized at the level of participants
- Participants in treatment with common mental health disorders, with the majority of participants having a diagnosis or clinical assessments indicating such a problem
- Any age groups; studies with child and youth populations, as well as adult patient populations
- Any studies of interventions where patient outcome data were given to therapists, patients, or both, on a regular basis for the duration of therapy
- Any primary care, out- or inpatient therapy, multidisciplinary mental health care, or other psychological therapy settings
- Studies where subsets of the data may qualify (fulfill criteria 1–4), e.g., three-armed RCTs where a portion of participants are relevant and can be extracted

### Exclusion Criteria

- Non-randomized design, including comparisons of assumed similar groups treated at different time periods, or benchmark studies
- Studies comparing MFS to other treatment options besides treatment as usual (TAU)
- Studies where the intervention arm also included other manual-based or otherwise defined interventions not available to both the intervention and control groups
- Studies of group therapy or couples’ therapy, and studies with more than 10% of the sample in drug/alcohol treatment or with dementia, learning disorders, or psychosis

### Information Sources and Search

The review updated the search from Rognstad et al. (2022) by conducting new searches in the Cochrane Central Registry of Controlled Trials (CENTRAL), American Psychology Associations’ PsychoInfo, Ovid MEDLINE and Epub Ahead of Print, In-Process, In-Data-Review & Other Non-Indexed Citations, Daily and Versions, and Excerpta Medica Database (EMBASE). The last search was done on January 31, 2023. The search terms and strategy are provided in Online Appendix 1.

All references were added to the Covidence systematic review software (Veritas Health Innovation, Melbourne, Australia; available at www.covidence.org). Abstracts and full texts were screened independently by two reviewers. When disagreement occurred within a pair of reviewers, full-text articles were obtained, and disagreement was discussed until a consensus was reached.

### Data Extraction and Risk of Bias

All data extraction was done by two of the reviewing authors independently for each study. Agreements were reached in meetings between the reviewers to produce a final data set. Predefined process and implementation elements were extracted, along with outcome data (post-treatment measurement means, standard deviations, and the number of respondents). Any outcome data on quality of life or functioning, mental health symptoms or mental health measures were included.

Risk of bias was also assessed independently by two reviewers and discussions were held to reach a consensus. Risk of bias was assessed in accordance with Cochrane Collaboration’s risk of bias tool (Sterne et al., 2019) for the studies that could provide outcome data suitable for use in the meta-analysis.

### Coding Interventions

A coding manual inspired by the methods used by Engell et al. (2020) was created to identify the process and implementation elements included in the interventions. The coding manual included discrete implementation strategies per the ERIC taxonomy (Powell et al., 2015) and additional process and implementation elements from CP-FIT (Brown et al., 2019). A list of the a priori implementation elements is provided in Online Appendix 2. Further, the manual was developed through a data-driven process where articles were reviewed, and candidate process and implementation elements not covered in ERIC or CP-FIT were noted until no new elements emerged. Using SPSS, all discrete process and implementation elements were listed as variables and coded as present or absent based on information provided in the articles. In addition, study characteristics and data such as sample sizes, effect sizes, and variance measures were coded. All articles were coded by two of the reviewers in parallel. Any discrepancies were discussed in meetings between the reviewers until a consensus was reached. Two of the coders had prior experience in coding elements and effects from similar projects and participated in all meetings where discrepancies were discussed. As we found that several of the elements we searched for were not reported in many of the articles, corresponding authors for the included studies were contacted via email with a survey. Twelve authors completed the survey, which led to a more complete dataset.

### Analysis

Primarily, we present a descriptive overview of the reporting of the process and implementation elements. The frequency of the presence of the elements in the codebook is reported to indicate the extent to which they were used in the MFS studies.

Effect sizes were calculated for all outcome measurements where reporting allowed for it. When available, post-treatment measurement means, standard deviation, and number of respondents were used to calculate Cohen’s d and variance of this effect size. Alternatively, reported effect sizes were converted into Cohen’s d. All effect sizes that indicated a treatment outcome, either in symptoms/mental health or quality of life/functioning, were included, resulting in 67 effect sizes from 30 studies, based on a total sample of 13,807 participants. A three-level analysis in accordance with the procedure described by Assink and Wibbelink (2016) allowing for the use of several outcome measures in each study was applied, as implemented in a shiny app (https://github.com/ToreWentzel-Larsen/threelevel).

Analyses were done using identified process and implementation elements as potential moderators. The process and implementation elements were used as indicators to test the hypotheses proposed by CP-FIT. Some of the elements were used for more than one of the hypotheses. Based on these indicators, studies were scored dichotomously as either meeting the premise of the hypotheses or not. In accordance with the CP-FIT, these hypothesis variables were grouped under the three general propositions of the model regarding what would govern the effect of feedback interventions: capacity limitations, identity and culture, and behavioral induction. *Capacity limitations* consisted of elements related to ease of use, automatization of collection and synthesizing of data, and immediate access to the data presentations. *Identity and culture* consisted of elements such as whether the therapists volunteered to participate and the presence of a “local advocate” or “champion.” *Behavioral induction* consisted of whether the feedback data were used in supervision or discussion groups, the presence of a clinical support tool (CST) or other devices to indicate potential actions warranted by the feedback data, the presence of a typology of patient progress, marking of not-on-track patients, lights or color coding, or messages to induce action from the therapists in response to the data. In the final step, more granular moderator analyses were performed in which data on the 42 more detailed hypotheses proposed by CP-FIT were applied as moderators.

Any descriptions of the degree of therapists’ use of feedback and therapists’ attitudes toward feedback were noted. An overview of these findings is provided in the results section. We planned to apply therapists’ use of feedback and therapists’ attitudes toward feedback as moderators for effect and, if possible, consider a mediating role for these variables between process and implementation variables and clinical outcomes. However, the review process revealed a lack of reporting in this regard that we considered quantifiable.

## Results

### Study Selection and Characteristics

**Fig. 1:**
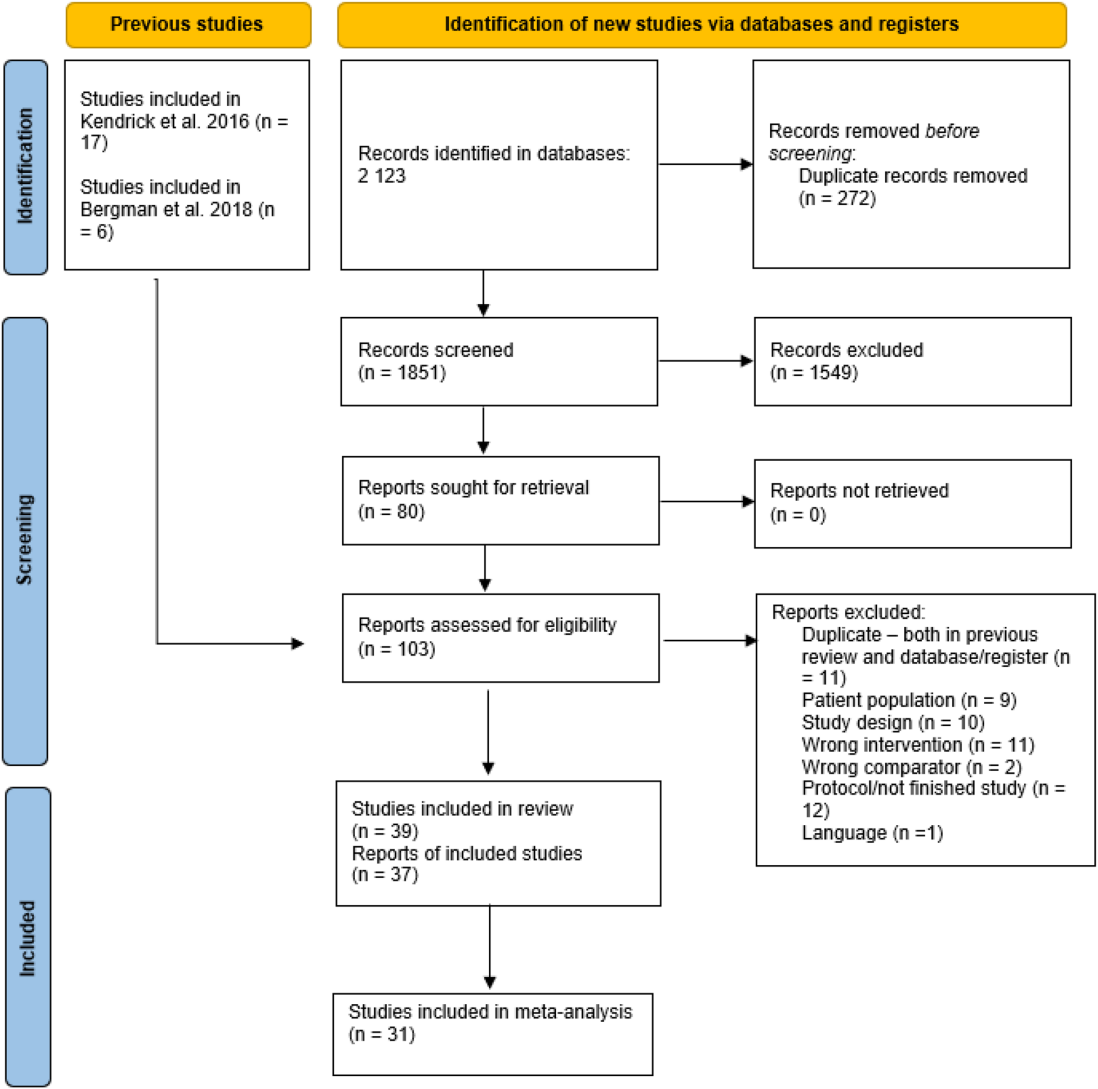
PRISMA flow diagram of the search and inclusion process

Figure 1 shows the flow diagram of the search and inclusion process (PRISMA flow diagram). The literature search updated the findings of Rognstad et al. (2022), resulting in 241 new abstracts, but after two reviewers independently screened all abstracts and four full-text articles, no further studies were included. Thus, the final sample consisted of the same 39 studies.

The 39 included studies were mainly from the United States (*k* = 22) and Europe (*k* = 16), and most studied the effects of either the Outcome Questionnaire (OQ; k = 13 studies) or the Partners for Change Outcome Management System (PCOMS; *k* = 12 studies).

### Risk of Bias in Identified Studies

All studies that allowed for the calculation of effect sizes were evaluated for risk of bias. All studies had high risk of bias due to the lack of blinding of personnel and participants (performance bias), while risk of bias was generally low or unclear in other areas (sequence generation, allocation concealment, blinding of outcome assessors, incomplete data/attrition bias, selective outcome reporting). Details on the within and across studies risk of bias can be found in Online Appendices 3 and 4.

### Descriptive Data

Data on the process and implementation elements were extracted from 39 studies. Outcome data could be calculated or extracted for 30 of the included studies. Any effect size indicating a treatment outcome, either in symptoms/mental health or quality of life/functioning, was included. This resulted in 67 effect sizes.

#### *Goal*s

A few studies indicated that they had engaged therapists in tailoring the design of the MFS (*k* = 5) and that the MFS had been tailored or adapted to the context (*k* = 5). Therapists did not seem to be involved in the choice of questionnaires or interviews for the feedback data. Most of the measures in the MFS were related to patients’ functioning/life circumstances during the previous week (*k* = 29) and/or symptoms/psychological distress (*k* = 25), while quite a few involved measures of alliance (*k* = 18). It is unclear whether the therapists’ sense of clinically relevant information informed the choice of these measures in any of the projects.

#### Data Collection and Analysis

There has been a tendency towards more automatization in the field in recent years. Thirteen of the studies had an electronic solution to gather data for the feedback system, and all of these were published post-2012. Fifteen of the studies reported that therapists were responsible for collecting the data; in twelve studies they did not have this responsibility, while the rest of the studies did not report on this. In about a third of the studies, data had to be transferred from paper surveys to electronic devices (*k* = 13).

#### Display

Most of the systems display graphs for patient progress (*k* = 31). Several of the systems gave indications of patients that were not-on-track (*k* = 24) or other typologies of patients (*k* = 3) e.g., by indicating patients above or below clinical cut-off scores.

#### Delivery

Feedback was given to therapists in a multitude of ways, either e-mailed (*k* = 3), on paper (*k* = 16), added to case records (*k* = 4), in a web application (9), or via an unreported delivery method.

#### Recipient Variables

In 14 studies, it was clearly stated that the therapists voluntarily participated in the feedback project. In one study, five sites allowed therapists to opt into the project, while it was obligatory at a sixth site. In the remaining studies, MFS was implemented at the institutional level (*k* = 10), or the studies did not provide any information on this.

#### Implementation Process

Only a few studies reported having a formal implementation plan *k* = (3) or that they had assessed readiness and/or identified barriers and facilitators for the implementation of MFS (*k* = 7). Likewise, few studies reported that they had provided training to leaders in implementation leadership or in supporting therapists’ use of MFS (*k* = 2).

Over half the studies described giving the feedback recipients training in using the MFS (*k* = 25) and reported providing training in the interpretation of the feedback reports (*k* = 25). Procedures for the use of the system or interpretation of the data were also manualized in most of the studies (*k* = 28). Half of the studies reported having technical assistance available for the users of the MFS (*k* = 20), and in six instances there was a local advocate/champion for the project.

#### Co-interventions

Only one study reported that the feedback data was also used for broader staff evaluation purposes. Four of the studies also implemented the clinical support tools (CST) associated with the Outcome Questionnaire and seven other studies had other systems to suggest or define suitable therapeutic action in response to feedback results.

#### Organization and Team Characteristics

Eight studies reported facilitating clinical supervision informed by feedback data, and in seven studies, feedback data was used in collegial group discussions. Two studies reported that training was provided to leaders in implementation leadership or in supporting therapists’ use of MFS.

### Therapists’ Use of Feedback

Twelve studies did not contain information on how therapists used the system and another nine reported explicitly that they had no procedures for monitoring use. In the latter group, some indicated an impression of ambivalence and lack of commitment (e.g., Hansson et al., 2013).

The remaining studies had either some form of quantifiable data or commented more qualitatively on the therapists’ feedback use (or lack thereof). We did not consider it meaningful to use these levels of therapists’ use as a moderator of effect as the studies reported this in very different ways—post-hoc self-reports, data tracking, surveys for clinicians to evaluate the MFS, or observations and comments from the researchers. Some of the researchers’ comments gave the impression that feedback was heavily underused, as the articles reported, for example, that “several indications that counselors’ adherence to the PCOMS intervention was low” (Cooper, M 2021, p. 29).

Lutz et al. (2021) measured the therapist-rated usefulness of feedback, therapist-rated negative effects of feedback, and therapist evaluation of the feedback system, but not therapist use of the MFS directly. In a related vein, Trudeau (2000) asked clinicians to complete the Provider Satisfaction Questionnaire and received both positive evaluations of the feedback system and reports of feelings that it was a waste of time. Kellybrew-Miller (2015) monitored adherence through integrity checklists after each session and 78% of the integrity checklists were completed correctly in the MFS condition, indicating correct implementation.

Several of the studies had post-hoc self-reports from therapists about feedback use and the level of commitment from therapists varied considerably. In two studies, about half of the therapists indicated that they had not used the feedback, while in several other studies about a third had no interaction with the feedback (*k* = 3). More use was found in two studies, namely 72% in Connolly Gibbons et al. (2015) and 89% in Chang et al. (2012). In other studies, therapists reported a degree of use, as in Lambert et al. (2001) where therapists generally reported using the feedback “at least to a moderate degree” (p. 62). In Amble et al. (2014, 2015), the therapists reported that they used the feedback to a large extent (4.47 on a scale of 1–5). Similarly, the therapists in McClintock et al. (2017) were asked how frequently they discussed feedback with clients and reported a mean rating of 4.67 on a 5-point Likert scale (1 = never, 5 = always).

Three studies had data to calculate an implementation index indicating the degree to which therapists used MFS (Bickman et al., 2016 [rural] [urban]; Janse et al., 2020). Bickman et al. (2016) used these data to provide evidence that increased use also increased effect. In Janse et al. (2020), cases with a high implementation index had significantly shorter treatment and a tendency towards larger symptom reduction (albeit not statistically significant) compared to those with a low implementation index. Two other studies also used implementation or therapist use as a moderator in their analysis. Although they did not find a significant beneficial effect of the feedback in their full sample, de Jong et al. (2012) found that not-on-track cases had a significant positive effect when therapists indicated that they used the feedback. Brattland et al. (2018) tested a Time × Condition interaction and found MFS to have increasingly more effect with longer implementation time. In other words, the difference in post-treatment distress between conditions became larger for each month of the 4-year-long trial in favor of the MFS condition compared to TAU (Brattland et al., 2018).

### Therapists’ Attitudes Towards Measurement and Feedback Systems

Most studies presented no data on therapists’ attitudes towards measurement or feedback systems, nor did they report anything about readiness for or user involvement prior to the implementation of the MFS intervention. Primarily, we were interested in data on therapists’ attitudes measured before the project started. Therapists’ attitudes were reported in two of the studies. McClintock et al. (2017) measured therapists’ beliefs about the effectiveness of therapy with or without MFS. In this study, the therapists believed that MFS-bolstered therapy would be more effective than TAU (4.50 vs. 3.83 on a 5-point Likert scale). Beliefs about MFS’ increased effectiveness were not used as a moderator in the study. de Jong et al. (2012) measured both perceived validity and commitment to using feedback. The correlation between perceived validity and commitment to using feedback was strong (r = .70). Surprisingly, a significant interaction was found between commitment and feedback in a negative direction, indicating that higher commitment led to a slower rate of change among the patients in cases where the therapist received feedback.

Lutz et al. (2021) gathered responses to the survey Therapist Attitude Toward and Confidence in Using Feedback after the project ended. This indicator of overall attitude to the use of MFS was significantly associated with better treatment outcomes on most of the outcome measures in the study. Lutz et al. (2015) found a larger effect size when therapists were satisfied with the MFS project than when they were not.

Several studies had post hoc surveys or interviews with the therapists to gauge satisfaction or perceived usefulness of the intervention (*k* = 8), while others only provided anecdotal or general statements about acceptance (*k* = 2). We deemed that the measures and interviews used were too heterogeneous for a comparison or summary to be sensible.

### Process and Implementation Elements as Moderators of MFS Effects

A significant overall effect size estimate for clinical outcomes was found favoring MFS over TAU (d = 0.14, 95% CI [0.08–0.21], p < 0.001). The data could indicate the presence/absence of 16 of CP-FIT’s 42 hypotheses. These were summed up in the three general CP-FIT propositions, and moderator analyses were performed to investigate whether variables related to CP-FIT’s three general propositions adjusted the effect of MFS. None of the groupings of variables that represented the three general propositions of CP-FIT significantly moderated MFS effects: *Capacity limitations* (slope = − 0.013, 95% CI [− 0.033 to 0.007], p = 0.185); *Identity and culture* (slope = − 0.014, 95% CI [− 0.04 to 0.012], p = 0.284); *Behavioral induction* (slope = − 0.028, 95% CI [− 0.058 to 0.002], p = 0.067). The moderation of MFS effects from the more specific CP-FIT hypotheses was also tested separately, but these were non-significant (see Online Appendix 5 for a complete list of CP-FIT hypotheses for which our data set had variance that allowed for testing of moderation).

### Heterogeneity and Risk of Reporting Bias

A modified Egger test (Egger et al., 1997; Marengo & Montag, 2020) was performed to investigate the heterogeneity among effect sizes. Significant heterogeneity was found (Q = 113.327, df = 65, p < 0.001), and a likelihood ratio test showed significant variance on the between-study level (SE = 0.12, p < 0.001). This suggests that between-study characteristics are likely to impact the overall effect estimate synthesizing effect sizes comparing MFS to TAU. The current review was initiated based on the proposition that much of the observed heterogeneity in the studies may be due to clinical differences and variation in implementation, as much as potential publication bias.

## Discussion

This review systematically mapped theoretically informed process and implementation elements of MFS across the literature (Brown et al., 2019; Engell et al., 2023). Identified elements included aspects related to data collection and analysis, display, delivery, recipient variables, context, co-interventions, and organization and team characteristics. We found only a few studies reporting that therapists were involved in tailoring the design of feedback systems. The measures used in feedback systems were mainly related to patients’ functioning, life circumstances, symptoms, psychological distress, and alliance, but it is unclear whether end users or therapists had any influence on the choice of outcome data. The application of user-centered design principles has been proposed to close the “research–practice gap” (Lyon & Koerner, 2016) and this is clearly lacking in most MFS studies thus far.

Data collection was done quite evenly via surveys distributed electronically or in pen and paper format, and in a large part of the studies, the therapists themselves were responsible for the data collection. In one study, the use of tablet surveys rather than pen-and-paper surveys was associated post hoc with greater implementation and positive effects of MFS (Bickman et al., 2016). Most systems displayed graphs for patient progress and indicated patients who were not on track.

Therapists voluntarily participated in feedback projects in a minority of the studies, and there was limited reporting on implementation plans, readiness assessments, and training for leaders in implementing feedback systems. In a clear minority of studies, feedback data were used for clinical supervision or group discussions. Overall, the findings highlight the need for more research on the implementation and effectiveness of feedback systems in mental health services. Despite searching in the studies for different implementation strategies, determinants, competencies, and other relevant parts of the implementation processes, most of the implementation elements in the identified articles reflect common implementation strategies (Waltz et al., 2015). One exception was therapist attitudes towards MFS which was reported in two studies. However, data from only two studies did not allow statistical testing of how attitudes were associated with effects.

Neither the moderator analyses used to test CP-FIT’s three general propositions nor those used to test the more specific hypotheses of the model had any significant results. In fact, the Behavioural induction proposition was close to significant in the opposite direction than proposed by the model. Most of the studies provided evidence of effect, or lack thereof, for different systems without identifying the factors likely to influence success. Although pragmatism may have its place, going forward, there is, as Kurt Lewin (1943) famously put it, “nothing as practical as a good theory.” CP-FIT is one potential framework for predicting the effects of MFS, but our data do not support the three general propositions in CP-FIT. However, this may be explained by the lack of attention to process and implementation and/or lack of reporting in the included studies, indicated by the low frequencies of relevant variables reported. Many rational ideas and strategies have been applied in the field that may help overcome barriers reported by health professionals and hypothesized by feedback theory. Still, so far, they appear to be underused, and we could not statistically connect these with the clinical effects of the interventions.

Some of the hypotheses in CP-FIT may not apply to MFS implementation, and specific functions of MFS interventions may influence therapists in more ways than one. For example, automated data collection should reduce the burden on the therapists and hence reduce problems related to capacity limitation. Yet it may also be that by demanding therapists to be “hands-on” in collecting and analyzing data, engagement with feedback data increases as therapists are already closely involved. As such, those demands may function as a nudge toward feedback engagement, a common strategy to change behavior (Yoong et al., 2020). The processes and elements hypothesized by CP-FIT to predict feedback use and success are based on a comprehensive systematic review of qualitative research on feedback interventions (Brown et al., 2019). However, these hypotheses may have to be tested experimentally to provide clearer answers regarding their relevance in MFS interventions.

Fidelity would be a key implementation outcome (Lewis et al., 2019), but the current review finds fidelity monitoring to be scarce and lacking standard reporting methods in the field. Therapists’ use of MFS was only reported in a few of the examined studies. The studies that provided information about use indicated that about one-third to half of the therapists did not engage with the available feedback. This might be expected as self-report surveys from therapists have indicated that even those who acknowledge the potential benefits of MFS may perceive the barriers to outweigh the positives (Chung & Buchanan, 2019). In different ways, a few of the studies were able to show that increased use was associated with better outcomes (Bickman et al., 2011, 2016; de Jong et al., 2012; Janse et al., 2020). Also, one study observed significant improvement in the MFS condition over time and proposed that this may be explained by continuous implementation efforts (Brattland et al., 2018). Therapists’ attitudes toward MFS, feedback, or standardized measures were very heterogeneously reported, and thus did not allow for data synthesis. Amble et al. (2014) showed high self-reported use by therapists who mostly self-recruited to the MFS project and higher effect sizes (Cohen’s d = .32) compared to the overall effect estimate of the current meta-analysis (Cohen’s d = .14).

We found the degree of implementation to be quite rarely reported, which did not permit comparison or data synthesis across studies. As Bickman et al. (2016) pointed out, a minimum for MFS studies should be to calculate an implementation index reporting the proportion of surveys answered by the patients and the proportion of feedback reports looked at by therapists. Appropriate measures of the degree of implementation can help exclude type III errors where one interprets study results as evidence of lack of effect despite attempting to measure something that did not exist (Dobson & Cook, 1980), in this case, the effect from assumed MFS use in cases lacking sufficient implementation of the system. Even though the implementation index proposed by Bickman et al. (2016) is a crude measure of implementation that only shows the potential amount of feedback the therapist could have used, both questionnaire completion and viewing of feedback must be at some reasonable level before implementation can be successful. Still, this would only indicate success in three of the first steps in the CP-FIT cycle (2. Data collection and analysis, 3. Feedback, and 4. Interaction). Following CP-FIT, one would also warrant measures of implementation elements such as perception, verification, and acceptance of the feedback, and subsequent intentions based on this reception. For this, it could be useful to gather documentation of when the therapists have discussed the feedback (Lewis et al., 2019) either with the clients, colleagues, or supervisors. In the current review, acceptance proved sparsely reported in most studies, and those who did often reported more general attitudes to measurement or satisfaction with the research project. We suggest that MFS researchers also attempt to gather data on the frequency of clinical decisions that are informed by the feedback data, as this will give an indication of the overall success of the implementation of a feedback system, from goal setting, data collection, therapist and client reception, intentions, to therapeutical behavior.

Only one of the identified studies (Bickman et al., 2016) reported feedback data being used for staff performance evaluation or other quality registry purposes. This will likely change as precision medicine and artificial intelligence are already making their way into psychotherapy clinics (Flemotomos et al., 2022; Imel et al., 2019). The same data may be used for MFS and as administrative tools and, as such, become an integrated part of clinics. This may facilitate the creation of a “culture of feedback” (Miller et al., 2016) where user opinions are welcomed and impact the services provided. On the other hand, it may also cause resistance from healthcare providers who experience discomfort and worry that the data will be used for top-down control and limit service accessibility (Moran et al., 2012). To facilitate the former effect and reduce the latter, efforts may need to be made to increase therapists’ ownership over MFS. An increased focus on process and implementation elements may be necessary to increase compatibility and appeal and to get therapists on board, for example by engaging them in tailoring MFS to their needs and providing appropriate training and supervision in MFS use. Therapists will be more likely to use MFS if they are drawn to the information gained rather than pushed by external pressures (Hatfield & Ogles, 2004).

### Limitations

The quality of the data from original studies influences the quality of any systematic review. As the current review set out to investigate topics that were not the central tenet of the literature reviewed, there are clear limitations. The degree of implementation, as well as therapists’ use and attitudes, were only reported in a small fraction of the identified studies. It was difficult to test all CP-FIT propositions as the original studies may have underreported implementation details and were not designed to test CP-FIT. In most cases, they were not designed to test any hypotheses about process or implementation. Several aspects of CP-FIT could not be tested, as the design or reporting in the original studies did not prioritize this.

Although 12 of the corresponding authors answered the survey we sent out to get a more complete data set, it is still unclear whether low frequencies of several variables are due to underreporting or their absence in the studies. The field of implementation science and the reporting standards it has produced are still young (see Rudd et al., 2020 for a review of reporting standards), and some of the studies date back to a time when, in general, publications devoted little space to implementation strategies and details. Also, in the newer studies, implementation aspects were likely not the focus of articles reporting outcomes from MFS interventions.

### Implications

Future studies on MFS should focus more on the processual aspects of MFS and implementation, both to increase the reach and effectiveness of MFS and to uncover the mechanisms for MFS’ success. Lack of reporting of process and implementation elements is a potential problem, and the mechanisms behind effective MFS interventions may be better charted if this improves in future studies.

It should be noted that several single studies pointed to increased use of and commitment to using MFS as being advantageous for clinical outcomes. This is likely to be promoted through well-planned and well-conducted implementation, even though the current review fails to link these elements to better outcomes. Future studies should aim to empirically test hypotheses regarding the process and implementation elements that are likely to influence the use of MFS as we currently cannot provide clear answers.

## Supplementary Information

Below is the link to the electronic supplementary material.

Supplementary file1 (DOCX 18 kb)

Supplementary file2 (DOCX 22 kb)

Supplementary file3 (DOCX 40 kb)

Supplementary file4 (DOCX 21 kb)

Supplementary file5 (DOCX 19 kb)

Supplementary file6 (DOCX 25 kb)
