# Supplementary material for: Process and Implementation Elements of Measurement Feedback Systems: A Systematic Review
Source: Adm Policy Ment Health. 2023 Dec 28;52(1):74–87. doi: 10.1007/s10488-023-01325-3 (PMC11703878; doi:10.1007/s10488-023-01325-3)
Supplement: Supplementary file 1 — Supplementary file1 (DOCX 18 kb) [file 10488_2023_1325_MOESM1_ESM.docx]

**Appendix 1. Search terms and strategy**

Database: Cochrane Central Register of Controlled Trials (CENTRAL)

Date Run: 30/01/2023 10:17:38

Search Strategy:

--------------------------------------------------------------------------------

#1 ((psychiatri* or psychotherapist* or therapist*) and ((client* or patient* or outpatient*) near (feedback or feed-back))):ti,ab,kw 326

#2 ((psychiatri* or psychotherapist* or therapist*) and (patient-reported near (outcome* or progress))):ti,ab,kw 314

#3 ((“psychotherapeutic outcome*” or “treatment outcome*”) near (feedback or feed-back or (patient-reported near (information or outcome* or progress)))):ti,ab,kw 58

#4 ((physician or “primary care” or “general practi*”) and ((client* or patient* or outpatient*) near (feedback or feed-back or progress))):ti,ab,kw 693

#5 MeSH descriptor: [Mental Disorders] explode all trees 83362

#6 MeSH descriptor: [Mental Health] explode all trees 2007

#7 MeSH descriptor: [Psychological Phenomena] explode all trees 100635

#8 ((#1 or #2 or #3) or (#4 and (#5 or #6 or #7))) with Cochrane Library publication date Between May 2022 and Jan 2023 71

***************************

Database: Cochrane Central Register of Controlled Trials (CENTRAL) – update 2023

Date Run: 26/01/2023 15:40:11

Search Strategy:

--------------------------------------------------------------------------------

#1 ((psychiatri* or psychotherapist* or therapist*) and ((client* or patient* or outpatient*) near (feedback or feed-back))):ti,ab,kw 326

#2 ((psychiatri* or psychotherapist* or therapist*) and (patient-reported near (outcome* or progress))):ti,ab,kw 314

#3 (psychotherapeutic outcome* and (feedback or feed-back or (patient-reported and (information or outcome* or progress)))):ti,ab,kw 44

#4 ((physician or “primary care” or “general practi*” or clinician*) and ((client* or patient* or outpatient*) near (feedback or feed-back or progress))):ti,ab,kw 1012

#5 MeSH descriptor: [Physician-Patient Relations] this term only 1480

#6 MeSH descriptor: [Mental Disorders] explode all trees 83362

#7 MeSH descriptor: [Mental Health] explode all trees 2007

#8 ((#1 or #2 or #3) or ((#4 or #5) and (#6 or #7))) 921

#9 (adolesc* or child* or paediatric* or pediatric*):so 46671

#10 (CAMHS or child* or boy* or girl* or infant* or juvenil* or minors or paediatric* or pediatric* or school* or kindergarten or nursery or adolesc* or pubert* or pubescen* or pupil* or teen* or young or youth* or student* or undergrad* or graduate or graduates or university or college or campus):ti 167023

#11 (#9 or #10) 184153

#12 (#8 and #11) 62

#13 ((feedback or feed-back) near (CAMHS or child* or boy* or girl* or infant* or juvenil* or minors or paediatric* or pediatric* or school* or kindergarten or nursery or adolesc* or pubert* or pubescen* or pupil* or teen* or young or youth* or student* or undergrad* or graduate or graduates or university or college or campus)) 2130

#14 (#13 and (#6 or #7)) 218

#15 (#12 or #14) with Cochrane Library publication date Between May 2022 and Jan 2023 10

***************************

Database: Embase Classic+Embase <1947 to 2023 January 26>

Search Strategy:

--------------------------------------------------------------------------------

1 ((physician* or psychiatri* or psychotherapist* or psychotherapy or therapist* or primary care or general practi* or clinician*) and ((client* or patient* or outpatient*) adj5 (feedback or feed-back))).ti,ab,kw. (4402)

2 ((psychiatri* or psychotherapist* or psychotherapy or therapist*) and (patient-reported adj5 (outcome* or progress))).ti,ab,kw. (980)

3 (psychotherapeutic outcome* and (feedback or feed-back or (patient-reported and (information or outcome* or progress)))).ti,ab,kw. (7)

4 ((physician or "primary care" or "general practi*" or clinician*) and ((client* or patient* or outpatient*) adj5 (feedback or feed-back or progress))).ti,ab,kw. (4976)

5 ((CORS or CSRS or OQ or PCOMS or SRS) and (feedback or feed-back or fed back)).ti,ab,kw. (201)

6 or/1-5 (7326)

7 (CAMHS or child* or boy* or girl* or infant* or juvenil* or minor* or paediatric* or pediatric* or school* or kindergarten or nursery or adolesc* or pubert* or pubescen* or pupil* or teen* or young or youth* or student* or undergrad* or graduate or graduates or college or campus or university).ti,ab,kw. (5563433)

8 6 and 7 (1708)

9 ((feedback or feed-back) adj5 (CAMHS or child* or boy* or girl* or infant* or juvenil* or minors or paediatric* or pediatric* or school* or kindergarten or nursery or adolesc* or pubert* or pubescen* or pupil* or teen* or young or youth* or student* or undergrad* or graduate or graduates or university or college or campus)).ti,ab,kw. (9559)

10 doctor patient relation/ (118308)

11 exp mental disease/ (2702922)

12 mental health/ (196886)

13 or/10-12 (2898758)

14 9 and 13 (1032)

15 8 or 14 (2712)

16 crossover-procedure/ or double-blind procedure/ or randomized controlled trial/ or single-blind procedure/ or (random* or factorial* or crossover* or cross over* or placebo* or (doubl* adj blind*) or (singl* adj blind*) or assign* or allocat* or volunteer*).tw. (2850061)

17 15 and 16 (531)

18 retracted article/ (15322)

19 erratum/ (258232)

20 (retraction or retracted or withdrawn or errata or erratum or correction).ti. (281481)

21 or/18-20 (330402)

22 15 and 21 (2)

23 17 or 22 (532)

24 (2023* or 2022*).yr,dc. (2420759)

25 23 and 24 (52)

***************************

Database: Ovid MEDLINE(R) ALL <1946 to January 25, 2023>

Search Strategy:

--------------------------------------------------------------------------------

1 ((physician* or psychiatri* or psychotherapist* or psychotherapy or therapist* or primary care or general practi* or clinician*) and ((client* or patient* or outpatient*) adj5 (feedback or feed-back))).ti,ab,kw. (2253)

2 ((psychiatri* or psychotherapist* or psychotherapy or therapist*) and (patient-reported adj5 (outcome* or progress))).ti,ab,kw. (522)

3 (psychotherapeutic outcome* and (feedback or feed-back or (patient-reported and (information or outcome* or progress)))).ti,ab,kw. (5)

4 ((physician or "primary care" or "general practi*" or clinician*) and ((client* or patient* or outpatient*) adj5 (feedback or feed-back or progress))).ti,ab,kw. (2569)

5 ((CORS or CSRS or OQ or PCOMS or SRS) and (feedback or feed-back or fed back)).ti,ab,kw. (129)

6 or/1-5 (3833)

7 (CAMHS or child* or boy* or girl* or infant* or juvenil* or minor* or paediatric* or pediatric* or school* or kindergarten or nursery or adolesc* or pubert* or pubescen* or pupil* or teen* or young or youth* or student* or undergrad* or graduate or graduates or college or campus or university).ti,ab,kw. (3965874)

8 6 and 7 (768)

9 ((feedback or feed-back) adj5 (CAMHS or child* or boy* or girl* or infant* or juvenil* or minors or paediatric* or pediatric* or school* or kindergarten or nursery or adolesc* or pubert* or pubescen* or pupil* or teen* or young or youth* or student* or undergrad* or graduate or graduates or university or college or campus)).ti,ab,kw. (6685)

10 Physician-Patient Relations/ (75968)

11 exp Mental Disorders/ (1408996)

12 Mental Health/ (57978)

13 or/10-12 (1516195)

14 9 and 13 (538)

15 8 or 14 (1282)

16 randomized controlled trial.pt. (585185)

17 controlled clinical trial.pt. (95168)

18 (randomized or randomised).ab. (706302)

19 placebo.ab. (235406)

20 clinical trials as topic.sh. (200789)

21 randomly.ab. (401084)

22 trial.ti. (278628)

23 or/16-22 (1545928)

24 retracted publication.pt. (12916)

25 "retraction of publication".pt. (13348)

26 comment.pt. (993727)

27 published erratum.pt. (128027)

28 (retraction or retracted or withdrawn or errata or erratum or correction).ti. (155477)

29 or/24-28 (1202900)

30 15 and 23 (248)

31 15 and 29 (2)

32 30 or 31 (250)

33 (2023* or 2022*).yr,dt,ed,ep. (2188943)

34 32 and 33 (20)

***************************

Database: APA PsycInfo <1806 to January Week 3 2023>

Search Strategy:

--------------------------------------------------------------------------------

1 ((physician* or psychiatri* or psychotherapist* or psychotherapy or therapist* or primary care or general practi* or clinician*) and ((client* or patient* or outpatient*) adj5 (feedback or feed-back))).ti,ab. (1107)

2 ((psychiatri* or psychotherapist* or psychotherapy or therapist*) and (patient-reported adj5 (outcome* or progress))).ti,ab. (156)

3 (psychotherapeutic outcome* and (feedback or feed-back or (patient-reported and (information or outcome* or progress)))).ti,ab. (7)

4 ((physician or "primary care" or "general practi*" or clinician*) and ((client* or patient* or outpatient*) adj5 (feedback or feed-back or progress))).ti,ab. (911)

5 ((CORS or CSRS or OQ or PCOMS or SRS) and (feedback or feed-back or fed back)).ti,ab. (127)

6 or/1-5 (1723)

7 (CAMHS or child* or boy* or girl* or infant* or juvenil* or minor* or paediatric* or pediatric* or school* or kindergarten or nursery or adolesc* or pubert* or pubescen* or pupil* or teen* or young or youth* or student* or undergrad* or graduate or graduates or college or campus or university).ti,ab. (1882388)

8 6 and 7 (419)

9 exp Therapeutic Processes/ (81888)

10 COUNSELING/ (25250)

11 PSYCHOTHERAPY/ (57760)

12 exp PSYCHOTHERAPEUTIC OUTCOMES/ (5565)

13 TREATMENT OUTCOMES/ (39005)

14 THERAPISTS/ (12134)

15 or/9-14 (195041)

16 FEEDBACK/ (21051)

17 ((feedback or feed-back) adj5 (CAMHS or child* or boy* or girl* or infant* or juvenil* or minors or paediatric* or pediatric* or school* or kindergarten or nursery or adolesc* or pubert* or pubescen* or pupil* or teen* or young or youth* or student* or undergrad* or graduate or graduates or university or college or campus)).ti,ab. (7891)

18 15 and 17 (164)

19 16 and 17 (3123)

20 8 or 18 or 19 (3611)

21 clinical trials.sh. (12131)

22 (randomi#ed or randomi#ation or randomi#ing).ti,ab,id. (104728)

23 (RCT or at random or (random* adj3 (assign* or allocat* or control* or crossover or cross-over or design* or divide* or division or number))).ti,ab,id. (113931)

24 (control* and (trial or study or group) and (placebo or waitlist* or wait* list* or ((treatment or care) adj2 usual))).ti,ab,id,hw. (33591)

25 ((single or double or triple or treble) adj2 (blind* or mask* or dummy)).ti,ab,id. (28723)

26 trial.ti. (36743)

27 placebo.ti,ab,id,hw. (43513)

28 or/21-27 (192762)

29 20 and 28 (396)

30 (retraction or retracted or withdrawn or errata or erratum or correction).ti. (19978)

31 20 and 30 (18)

32 29 or 31 (411)

33 (2022* or 2023*).yr,up. (210823)

34 32 and 33 (28)

***************************

Database: APA PsycInfo <1806 to January Week 4 2023>

Search Strategy:

--------------------------------------------------------------------------------

1 COUNSELING/ (25258)

2 PSYCHOTHERAPY/ (57780)

3 PSYCHOTHERAPEUTIC OUTCOMES/ (5566)

4 TREATMENT OUTCOMES/ (39021)

5 patient reported outcome measures/ (629)

6 THERAPISTS/ (12138)

7 "3310".cc. (80402)

8 or/1-7 (171011)

9 FEEDBACK/ (21071)

10 (feedback or feed-back).ti,id. (25357)

11 or/9-10 (29219)

12 8 and 11 (848)

13 ((physician* or psychiatri* or psychotherapist* or therapist* or primary care or general practi*) and ((client* or patient* or outpatient*) adj5 (feedback or feed-back))).ti,ab,id. (898)

14 ((physician* or psychiatri* or psychotherapist* or therapist* or primary care or general practi*) and (patient reported adj3 (information or outcome*))).ti,ab,id. (439)

15 (psychotherapeutic outcome* and (feedback or feed-back or (patient reported adj3 (information or outcome*)))).ti,ab,id. (14)

16 or/12-15 (2009)

17 TREATMENT EFFECTIVENESS EVALUATION/ (27344)

18 CLINICAL TRIALS/ (12131)

19 MENTAL HEALTH PROGRAM EVALUATION/ (2305)

20 randomly.ab. (81812)

21 randomi#ed.ti,ab,id. (101836)

22 (control* adj3 (trial or study or group*1)).ti,ab,id. (163461)

23 ("0300" or "2100").md. (56570)

24 (waitlist* or (wait* and list* and (control* or group))).ti,ab,id. (8105)

25 (treatment as usual or TAU or usual care or care as usual).ti,ab,id. (21386)

26 or/17-25 (329654)

27 16 and 26 (524)

28 (3 or 4) and 11 (366)

29 27 or 28 (725)

30 (2023* or 2022*).up,ch. (293932)

31 29 and 30 (62)

***************************
