# Supplementary material for: Process and Implementation Elements of Measurement Feedback Systems: A Systematic Review
Source: Adm Policy Ment Health. 2023 Dec 28;52(1):74–87. doi: 10.1007/s10488-023-01325-3 (PMC11703878; doi:10.1007/s10488-023-01325-3)
Supplement: Supplementary file 3 — Supplementary file3 (DOCX 40 kb) [file 10488_2023_1325_MOESM3_ESM.docx]

| **Appendix 3. Cochrane Risk of Bias Comparison**  **Individual Risk of Bias for studies included in the meta-analysis** | | | | | | | |
| --- | --- | --- | --- | --- | --- | --- | --- |
| Author(s) | Sequence generation | Allocation concealment | Blinding of participants for all outcomes | Blinding of personnel for all outcomes | Blinding of outcome assessors for all outcomes | Incomplete outcome data for all outcomes | Selective outcome reporting |
| Amble et al. 2014 | 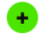 | 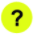 | 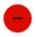 | 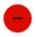 | 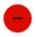 | 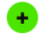 | 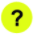 |
| Bastiaansen et al. 2021 | 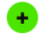 | 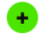 | 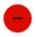 | 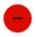 | 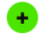 | 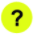 | 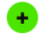 |
| Bickman et al. 2011 | 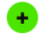 | 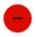 | 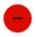 | 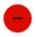 | 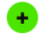 | 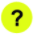 | 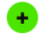 |
| Bovendeerd et al. 2021 | 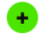 | 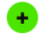 | 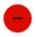 | 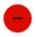 | 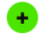 | 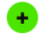 | 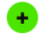 |
| Brattland et al. 2018 | 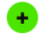 | 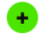 | 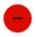 | 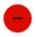 | 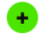 | 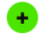 | 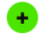 |
| Chang et al. 2012 & Yeung et al. 2012 | 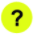 | 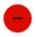 | 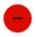 | 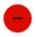 | 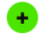 | 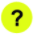 | 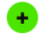 |
| Cooper et al. 2019 | 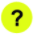 | 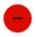 | 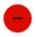 | 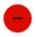 | 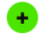 | 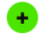 | 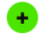 |
| De Jong et al. 2012 | 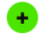 | 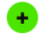 | 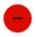 | 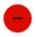 | 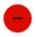 | 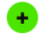 | 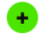 |
| De Jong et al. 2014 | 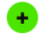 | 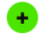 | 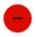 | 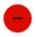 | 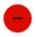 | 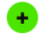 | 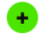 |
| Delgadillo et al. 2018 | 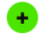 | 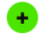 | 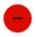 | 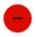 | 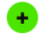 | 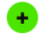 | 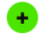 |
| Duncan et al. 2021 | 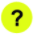 | 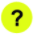 | 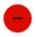 | 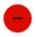 | 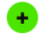 | 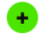 | 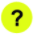 |
| Errazuriz et al. 2018 | 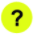 | 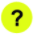 | 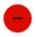 | 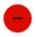 | 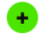 | 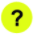 | 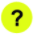 |
| Hansson et al. 2013 | 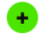 | 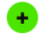 | 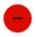 | 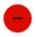 | 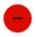 | 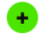 | 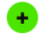 |
| Hawkins et al. 2004 | 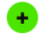 | 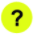 | 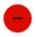 | 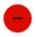 | 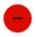 | 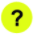 | 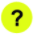 |
| Janse et al. 2020 | 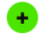 | 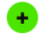 |  |  |  |  |  |
| Kelleybrew-Miller 2017 |  |  |  |  |  |  |  |
| Kendrick et al. 2017 |  |  |  |  |  |  |  |
| Lambert et al. 2001 |  |  |  |  |  |  |  |
| Lester et al. 2012 |  |  |  |  |  |  |  |
| Lutz et al. 2015 |  |  |  |  |  |  |  |
| Lutz et al. 2021 |  |  |  |  |  |  |  |
| Melendez 2002 |  |  |  |  |  |  |  |
| Murphy et al. 2012 |  |  |  |  |  |  |  |
| Reese et al. 2009 study 1 |  |  |  |  |  |  |  |
| Reese et al. 2009 study 2 |  |  |  |  |  |  |  |
| Rise et al. 2016 |  |  |  |  |  |  |  |
| Schottke et al. 2019 |  |  |  |  |  |  |  |
| She et al. 2018 |  |  |  |  |  |  |  |
| Simon et al. 2012 |  |  |  |  |  |  |  |
| Trudeau et al. 2000 |  |  |  |  |  |  |  |
| Whipple et al. 2003 |  |  |  |  |  |  |  |
|  |  |  |  |  |  |  |  |

*Note.* Red = high Risk of Bias, yellow = unclear Risk of Bias, and green = low Risk of Bias*.*
