# Supplementary material for: Process and Implementation Elements of Measurement Feedback Systems: A Systematic Review
Source: Adm Policy Ment Health. 2023 Dec 28;52(1):74–87. doi: 10.1007/s10488-023-01325-3 (PMC11703878; doi:10.1007/s10488-023-01325-3)
Supplement: Supplementary file 4 — Supplementary file4 (DOCX 21 kb) [file 10488_2023_1325_MOESM4_ESM.docx]

**Appendix 4. Risk of Bias summarized across studies**
