# Supplementary material for: Process and Implementation Elements of Measurement Feedback Systems: A Systematic Review
Source: Adm Policy Ment Health. 2023 Dec 28;52(1):74–87. doi: 10.1007/s10488-023-01325-3 (PMC11703878; doi:10.1007/s10488-023-01325-3)
Supplement: Supplementary file 5 — Supplementary file5 (DOCX 19 kb) [file 10488_2023_1325_MOESM5_ESM.docx]

**Appendix 5. CP-FIT hypotheses tested**

| Hypothesis: Feedback interventions are more effective when … | General propositions | Results moderator-analysis |
| --- | --- | --- |
| 4. Conducted by recipients: … They do not require the recipient to collect or analyse the clinical performance data. | Capacity limitations | Estimated difference - 0.050  lower - 0.176  upper 0.076  p.value 0.430 |
| 5. Automation: … They collect and analyse data automatically rather than manually. | Capacity limitations | Estimated difference - 0.015  lower - 0.142  upper 0.112  p.value 0.819 |
| 8. Performance level: … They communicate recipients’ current performance has room for improvement | Identity and culture  Behavioural induction | Estimated difference - 0.070  lower - 0.204  upper 0.063  p.value 0.296 |
| 11. Timeliness: … They use recent data to calculate recipients’ current performance | Identity and culture  Behavioural induction | Estimated difference 0.116  lower - 0.065  upper 0.298  p.value 0.205 |
| 13. Benchmarking: … They compare recipients’ current performance to that of other health professionals, organisations or regions. | Capacity limitations  Identity and culture | Estimated difference - 0.090  lower - 0.228  upper 0.047  p.value 0.194 |
| 18. Active delivery: … They “push” feedback messages to recipients rather than requiring them to “pull”. (Except if solely delivered face-to-face, which increases 41. Cost) | Capacity limitations  Identity and culture | Estimated difference 0.362  lower - 0.144  upper 0.868  p.value 0.158 |
| 20. Feedback attitude: … They target health professionals with positive beliefs about feedback. | Capacity limitations  Identity and culture | Estimated difference - 0.062  lower - 0.189  upper 0.064  p.value 0.329 |
| 22. Knowledge and skills in clinical topic: … They target health professionals with greater capability in the clinical topic under focus. | Capacity limitations  Behavioural induction | Estimated difference - 0.048  lower - 0.185  upper 0.088  p.value 0.485 |
| 26. Leadership support: … They are supported by senior managers. | Capacity limitations  Identity and culture | Estimated difference - 0.143  lower - 0.390  upper 0.104  p.value 0.251 |
| 27. Champions: … They are supported by individuals in the organisation dedicated to making it a success. | Capacity limitations  Identity and culture | Estimated difference 0.029  lower - 0.139  upper 0.197  p.value 0.730 |
| 34. Peer discussion: … They encourage recipients discuss their feedback with peers. | Capacity limitations  Identity and culture | Estimated difference 0.078  lower - 0.079  upper 0.236  p.value 0.324 |
| 35. Problem solving: … They help recipients identify and develop solutions to reasons for suboptimal performance (or support recipients to do so). | Capacity limitations  Identity and culture  Behavioural induction | Estimated difference - 0.102  lower - 0.220  upper 0.015  p.value 0.086 |
| 36. Action planning: … They provide solutions to suboptimal performance (or support recipients to do so). | Capacity limitations  Behavioural induction | Estimated difference - 0.102  lower - 0.220  upper 0.015  p.value 0.086 |
| 38. Adaptability: … They are tailored to the specific needs of the health care organisation and its staff. | Capacity limitations  Identity and culture | Estimated difference - 0.081  lower - 0.222  upper 0.061  p.value 0.260 |
| 39. Training and support: … They provide training and support regarding feedback | Capacity limitations  Behavioural induction | Estimated difference - 0.105  lower - 0.222  upper 0.012  p.value 0.079 |
| 42. Ownership: … Recipients feel they “own” it, rather than it has been imposed on them | Identity and culture | Estimated difference 0.008  lower - 0.152  upper 0.168  p.value 0.918 |
